# Culture but not gender modulates amygdala activation during explicit emotion recognition

**DOI:** 10.1186/1471-2202-13-54

**Published:** 2012-05-29

**Authors:** Birgit Derntl, Ute Habel, Simon Robinson, Christian Windischberger, Ilse Kryspin-Exner, Ruben C Gur, Ewald Moser

**Affiliations:** 1 MR Centre of Excellence, Medical University of Vienna, Vienna, Austria; 2 Institute for Clinical, Biological and Differential Psychology, Faculty of Psychology, University of Vienna, Vienna, Austria; 3 Department of Psychiatry, Psychotherapy, and Psychosomatics, RWTH Aachen University, Aachen, Germany; 4 Centre for Medical Physics and Biomedical Engineering, Medical University of Vienna, Vienna, Austria; 5 Department of Psychiatry, University of Pennsylvania Medical School, and the Philadelphia Veterans Administration Medical Center, Philadelphia, USA

**Keywords:** Culture, Gender, Emotion, Amygdala, fMRI

## Abstract

**Background:**

Mounting evidence indicates that humans have significant difficulties in understanding emotional expressions from individuals of different ethnic backgrounds, leading to reduced recognition accuracy and stronger amygdala activation. However, the impact of gender on the behavioral and neural reactions during the initial phase of cultural assimilation has not been addressed. Therefore, we investigated 24 Asians students (12 females) and 24 age-matched European students (12 females) during an explicit emotion recognition task, using Caucasian facial expressions only, on a high-field MRI scanner.

**Results:**

Analysis of functional data revealed bilateral amygdala activation to emotional expressions in Asian and European subjects. However, in the Asian sample, a stronger response of the amygdala emerged and was paralleled by reduced recognition accuracy, particularly for angry male faces. Moreover, no significant gender difference emerged. We also observed a significant inverse correlation between duration of stay and amygdala activation.

**Conclusion:**

In this study we investigated the “alien-effect” as an initial problem during cultural assimilation and examined this effect on a behavioral and neural level. This study has revealed bilateral amygdala activation to emotional expressions in Asian and European females and males. In the Asian sample, a stronger response of the amygdala bilaterally was observed and this was paralleled by reduced performance, especially for anger and disgust depicted by male expressions. However, no gender difference occurred. Taken together, while gender exerts only a subtle effect, culture and duration of stay as well as gender of poser are shown to be relevant factors for emotion processing, influencing not only behavioral but also neural responses in female and male immigrants.

## Background

Within the last decade the interdisciplinary field of cultural neuroscience investigating interrelations among culture, mind and the brain has increased tremendously cf. [1]. According to Chiao and Ambady [2], the main goal of cultural neuroscience is to investigate how much of the cultural variation observable in human behavior is traceable to cultural variation, including the biological and neural levels. Previous studies reported cultural differences in neural activation for a variety of cognitive functions including picture encoding [3], voting behavior [4], empathy [5,6], and self-representation [7,8].

Broad consensus exists that culture also asserts a significant impact on the neural correlates of face processing, particularly regarding activation of the amygdala, mostly reporting stronger or sustained activation to out-group faces e.g., [4,9-12]; for review see [1,13,14]. Emotional expressions (i.e., happy and fearful) have rarely been used in fMRI studies addressing culture effects, but play a special role in emotion processing: Moriguchi et al. [15] showed differences in the neural processing of fearful faces between Caucasian and Japanese subjects, with higher activation of the left amygdala in Caucasians. Chiao et al. [16] presented emotional expressions of Japanese and Caucasian actors to Japanese and Caucasian Americans and observed significantly elevated amygdala response to in-group expressions of fear in both groups, suggesting a specific sensitivity of the amygdala to optimally respond to facial expressions of fear specific to one’s own cultural group. Recently, Adams et al. [17] investigated the effect of direct vs. averted eye gaze during processing of fearful Caucasian and Japanese faces in Caucasian American and Japanese students. They observed elevated amygdala activation during the averted vs. direct gaze when expressions were posed by in-group posers, while out-group posers elicited stronger amygdala activation during direct vs. averted gaze. These findings reveal a meaningful role of culture in the processing of eye gaze and emotion, and highlight their interactive influences in neural processing. However, gender differences and the impact of gender of poser were not addressed in either study, although mixed samples were investigated and mixed stimuli presented.

Recently we reported bilateral amygdala response of Asian males and matched European Caucasian subjects to Caucasian emotional expressions [18]. We observed significantly stronger amygdala activation in Asian males that was inversely correlated with duration of stay. Moreover, a decreased recognition rate of disgust was demonstrated, probably indicating initial problems with a difficult emotion that may be shaped to a greater extent by cultural influences than the other basic emotions. As we only examined male participants, analysis of gender-by-culture interactions on amygdala response was not possible.

Gender differences in the activation of the amygdala during facial emotion processing have been documented frequently, albeit with findings being extremely heterogeneous: some studies showed stronger activation in females e.g., [19-21], some in males e.g., [22-24], and some studies report no significant activation difference e.g., [25-28]. In sum, these inconsistencies in the level of amygdala activation may be due to the emotions investigated and differences in the methodology employed (e.g., stimuli, task design, MR-methods, etc.) and results from a recent meta-analysis across 105 fMRI studies [29] point to a stronger activation of the right amygdala during facial emotion processing in males. However, studies specifically addressing the interaction of culture and gender on amygdala activation during emotion processing are still missing. More generally, none of the previous neuroimaging studies exploring cultural effects examined gender differences, though most relied on a mixed sample or used mixed stimuli [3-12,15-17].

Therefore, in the present study behavioral performance and amygdala activation were examined during an explicit emotion recognition task in female and male Asian immigrants (i.e., exchange students with a short residence time in Austria) and female and male Caucasian Europeans (Caucasian Austrians), allowing investigation of initial difficulties in emotion identification, their possible impact on amygdala response and analysis of potential gender-by-culture interactions regarding behavioral performance as well as neural activation patterns.

Based on previous findings e.g. [18,30-32], we hypothesized bilateral amygdala response in all subjects. However, according to findings on cultural differences in emotion processing behavior: e.g. [33-35]; neural correlates: e.g., [16,18], we posited a significant impact of ethnic group on behavioral and neural responses that affects the amygdala: we hypothesized stronger amygdala activation in the female Asian sample as they were out-group to the ethnic group of posers presented (i.e., Caucasians). Considering previous results on the significant effect of duration of stay on emotion recognition performance [36] and amygdala activation in male immigrants [18], we also expected a significant association between duration of stay and behavioral performance and amygdala activation in Asian females. Moreover, we aimed at further exploring the impact of gender and culture on amygdala activation, as previous studies, mentioned above, reported inconsistencies or did not address this issue.

## Results

### Behavioral data

Emotion recognition accuracy was 77.4% (SD = 18.5) on average for Asian and 90.8% (SD = 3.8) for European subjects. The repeated measures ANOVA revealed a significant emotion effect, F(5,220) = 9.359, p < .001, *η*² = .175, with highest accuracy for fearful expressions and lowest performance for disgust, a significant ethnic group effect, F(1,44) = 15.294, p < .001, *η*² = .258, with better performance of the Europeans, a significant gender of poser effect, F(1,44) = 6.715, p = .013, *η*² = .132, with higher scores for female posers, but no significant gender effect, F(1,44) = 2.339, p = .133, ns. Moreover, we observed a significant emotion-by-ethnic group interaction, F(5,220) = 6.690, p < .001, *η*² = .132, a significant emotion-by-gender of poser interaction, F(5,220) = 9.506, p < .001, *η*² = .178, and a significant emotion-by-gender of poser-by-ethnic group interaction, F(5,220) = 6.915, p < .001, *η*² = .136. No further interaction reached significance (all p-values > .154).

Disentangling the significant emotion-by-ethnic group interaction, we observed significant differences for anger (p < .001) and disgust (p < .001) that even remained significant after Bonferroni correction, indicating lower performance of Asians. Post-hoc analysis of the significant emotion-by-gender of poser interaction revealed that angry and disgusted expressions were better recognized in male posers (both p < .001) while fear was more accurately recognized in female posers (p = .009). For the other expressions no significant effect occurred (all p > .150). Looking at the three-way interaction (emotion-by-gender of poser-by-ethnic group) we noticed several significant differences, however after Bonferroni correction only those for female and male anger (both p < .001) as well as female and male disgust (female: p < .001; male: p = .001) remained significant indicating lower performance in the Asian sample.

Reaction times were 2.3 s (SD = 0.4 s) on average for Asians and 2.2 s (SD = 0.4 s) for Europeans. Analysis of reaction times revealed a significant emotion effect, F(5,220) = 20.737, p < .001, *η²*. = .320, with fastest response times to happy expressions, no significant ethnic group effect, F(1,44) = 2.712, p = .107, no significant gender of poser effect, F(1,44) = 0.065, p = .800, and no significant gender effect, F(1,44) = 1.735, p = .195. However, a significant emotion-by-gender of poser, F(5,220) = 8.096, p < .001, *η*² = .155, and a significant emotion-by-gender of poser-by-ethnic group interaction, F(5,220) = 8.690, p < .001, *η*² = .175, occurred. All other interactions were not significant (all p > .325).

**Figure 1:**
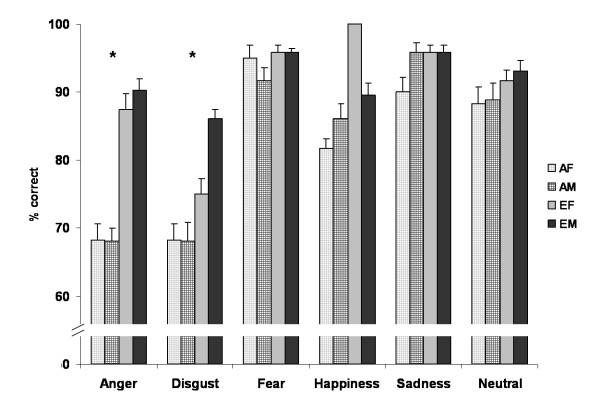
**Behavioral performance during the explicit emotion recognition task showing recognition accuracy with standard error of the mean (SEM) for all emotions.** Repeated measures ANOVA revealed a significant ethnic group effect (p < .001) with lower performance of the Asian participants, in particular for angry and disgusted faces marked with an asterisk

Figure 1 illustrates emotion recognition accuracy for Asians and Europeans.

Correlation analysis revealed neither a significant association between recognition performance and duration of stay, r(22) = 0.245, p = .112, nor between reaction time and duration of stay, r(22) = 0.018, p = .423.

### Functional data

**Figure 2:**
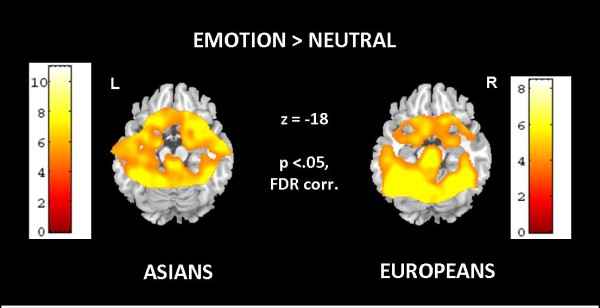
**Results of whole-slab analysis showing activation maps of random effects analysis for all emotions minus neutral on one axial slice comprising the amygdala for the Asian sample (left) and for the Caucasian European sample (right) (threshold: p < .05, FDR corrected).** Stronger bilateral amygdala response to emotional categories (minus neutral expressions) is visible in both groups.

Separate group analyses for Asian and European subjects showed bilateral amygdala activation to all presented emotions and neutral expressions, and direct comparison between emotional vs. neutral expressions revealed significantly stronger amygdala response to emotional faces. Besides amygdala activation, responses of bilateral fusiform gyrus, inferior occipital and frontal gyri, inferior and medial temporal regions, hippocampus and parahippocampal gyrus as well as brainstem and cerebellum emerged for all emotions and neutral expressions for Asians and Europeans, respectively (see Figure 2 and Table 1 for details). Regarding direct comparisons (Asians vs. Europeans, Females vs. Males), several differences occurred including the amygdala, although none of these survived an FDR corrected threshold.

**Table 1:** Results from the whole brain analysis showing activation in a widespread neural network during recognition of emotional faces in Asians and Europeans Inferior frontal gyrus (region) thresholded at p < .05, FDR corrected

**Contrast**	**MNI X Y Z**			**Cluster size**	**t-value**	**L/R**	**Region**
	**ASIANS -- EMOTION > NEUTRAL**							
	−4	−30	−10	1227	10.99	L	Brainstem	
	38	12	−30	536	8.60	R	Superior temporal gyrus	
	20	20	−22	105	6.46	R	Inferior frontal gyrus	
	−22	16	−26	575	5.15	L		
	−44	−36	−22	98	5.11	L	Fusiform gyrus	
	6	16	−12	37	4.35	R	Thalamus	
	−62	−6	−18	29	3.87	L	Middle temporal gyrus	
	−16	32	−22	20	3.70	L	Orbitofrontal gyrus	
	16	−4	−24	36	3.45	R	Amygdala	
	−20	−56	−16	23	3.44	L	Cerebellum	
	−22	0	−18	21	3.40	L	Amygdala	
	**EUROPEANS -- EMOTION > NEUTRAL**							
	34	−84	−14	2389	8.46	R	Inferior occipital gyrus	
	−44	−74	−14	738	8.43	L	Middle occipital gyrus	
	38	−64	−18	811	7.99	R	Fusiform gyrus	
	28	56	−34	47	4.51	R	Orbitofrontal gyrus	
	−18	−2	−16	44	4.43	L	Amygdala	
	22	0	−18	31	4.13	R	Amygdala	
	−16	22	−20	40	3.91	L	Inferior frontal gyrus	

MNI coordinates, cluster size (k > 20), t-values, laterality and region.

#### ROI analysis

The ROI analysis including the contrast estimates emotion > neutral for female and male posers, demonstrated a significant main effect of ethnic group, F(1,44) = 9.141, p = .004, *η*² = .175, with stronger activation in Asians but no significant effect of gender, F(1,44) = 0.197, p = .659, nor gender-by-ethnic group interaction, F(1,44) = 0.043, p = .837. Moreover, a significant gender of poser effect, F(1,44) = 5.332, p = .026, *η*² = .110, and a significant gender of poser-by-ethnic group interaction, F(1,44) = 4.491, p = .040, *η*² = .090, emerged. No laterality effect, F(1,44) = 0.861, p = .359, was observed and no further interaction was significant (all p > .280).

**Figure 3:**
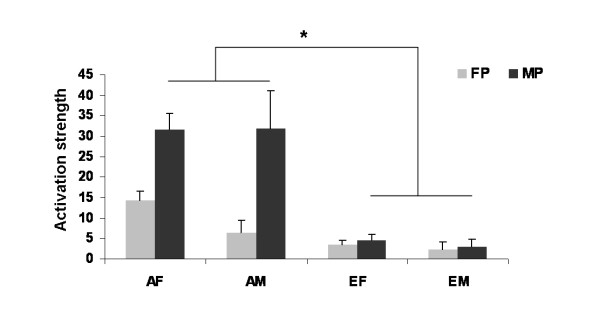
**Results from ROI analysis showing mean parameter estimates of the amygdala for the emotion > neutral contrast for female (FP) and male posers (MP) for Asian females (AF), Asian males (AM), European females (EF) and European males (EM), revealing significantly stronger amygdala activation in the Asian sample (p = .004).** Moreover, a significant gender of poser-by-ethnic group interaction (p = .040) emerged, indicating stronger activation of the Asian participants for male posers (p = .009) and a trend for stronger activation for female posers (p = .079)

Post-hoc analysis of the significant gender of poser-by-ethnic group interaction revealed a significant difference in amygdala activation for male posers (p = .009), with higher values in the Asian sample and a trend in the same direction for amygdala reactions to female posers (p = .079). Mean parameter estimates of Asian females and males and European females and males for the contrast emotion-neutral female poser and male poser are illustrated in Figure 3.

#### Correlations

Since we observed no significant laterality effect and no significant interaction, we performed correlation analyses using the mean parameters of amygdala activation. No significant correlation emerged between recognition accuracy and mean response of the amygdala, neither for the whole sample (p > .207), nor for Asians (p > .295) or Europeans (p > .102).

Analyzing the impact of duration of stay on mean amygdala activation across all Asian participants revealed a significant result, r(22) = −0.394, p = .031, indicating stronger amygdala response in those Asians with shorter duration of stay. Gender-specific analyses demonstrated a significant negative association for both, Asian females, r(11) = −0.550, p = .040, and Asian males, r(11) = −0.578, p = .025. To explore whether these correlations significantly differ we applied a Fisher’s Z transformation, which revealed no significant difference (Z = 0.497, Z < 1.65 which is the critical Z-value for alpha = 0.05). Using multiple hierarchical regression analyses, we assessed whether gender acts as a mediator variable. While gender alone showed no significant effect on amygdala activation (beta = −0.197, t = −0.920, p = .368), duration of stay showed a significant impact (beta = −0.532, t = −2.630, p = .016), but the interaction of gender and duration of stay remained not significant (beta = −0.386, t = −1.908, p = .071).

**Figure 4:**
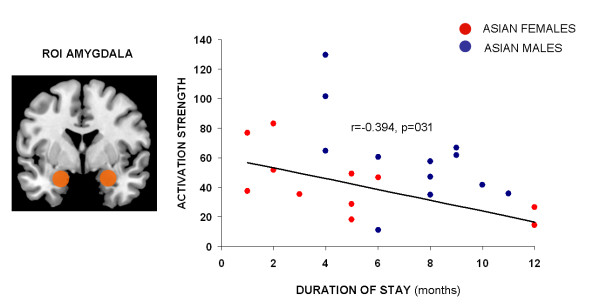
Correlation analysis between mean parameter estimates of the amygdala region and duration of stay in Europe (months) showing a significant negative association (r(22) = −0.394, p = .031) indicating stronger amygdala response in those Asian participants with shorter duration of stay and thus probably reflecting adaptation effects on the neural level.

Explorative analysis of impact of gender of poser on these correlations revealed a significant association between duration of stay and amygdala activation to male posers, r(22) = −0.343, p =.035, while no such correlation occurred for female posers, r(22) = −0.285, p = .188. Figure 4 illustrates the significant correlation between amygdala response and duration of stay in Asian females and males.

## Discussion

This study investigated the behavioral performance and neural activation during an explicit emotion recognition task to examine the impact of exposure to emotional expressions of a different, previously unfamiliar ethnic group in Asian female and male immigrants. In concordance with previous results from our lab on male immigrants [18] and our hypothesis, bilateral amygdala activation in both samples was observed, confirming the role of the amygdala as a 'relevance detector' [37]. The amygdala seems to be fundamental in emotion processing as a part of the underlying neural network although gender, socialization and cultural background seem to exert a certain impact on its activation.

### Amygdala activation and the alien-effect

In general, Asian females and males demonstrated significantly stronger neural response of the amygdala when asked to explicitly recognize the emotions presented by Caucasian actors, which reflects recent findings observed in an Asian male sample [18]. Moreover, significantly stronger amygdala activation was apparent during processing of male faces in the Asian sample, while only a trend emerged for female posers. Hence, recognizing emotions in Caucasian faces, and here particularly male faces, leads to stronger bilateral amygdala activation in Asians than in Caucasians who are viewing in-group faces. Interestingly, this effect is not specific to the gender of the rater since both female and male Asians responded similarly. This demonstrates that an alien-effect exists for female and male immigrants, a finding which extends our knowledge on ethnic group effects on (emotional) face processing e.g., [9-12,15,16,38]. The stronger amygdala activation might be related to novelty but also motivation and emotional learning e.g., [39] as Asian immigrants may want to assimilate with the host culture and thus emotional expressions of the foreign ethnic group exhibit a strong salient cue as shown previously [40,41]. Moreover, in our study we only presented faces with direct gaze and previous experiments indicated that the amygdala is sensitive to gaze direction e.g., [42,43]. Richeson and colleagues [44] even showed that race-related activity of the amygdala is modulated by eye-gaze. Very recently, Adams et al. [17] reported stronger amygdala activation to direct vs. averted gaze fear expressed by out-group posers in Caucasian American and Japanese students, while for in-group expressions averted vs. direct gaze yielded stronger amygdala response. Direct eye gaze has been shown to facilitate social categorization [45] but also communicates a range of intentions, amongst these also hostility [46] and has been shown to influence threat perceptions e.g., [47]. Hence, in our Asian participants’ elevated amygdala activation particularly to out-group male expressions might also be influenced by gaze direction, prompting a stronger need of fast evaluation. Additionally, several studies report greater attention to out-group faces apparent in larger amplitudes of the N100, the P200 and the P300 e.g., [48,49] probably reflecting an automatic vigilance effect in which attention is quickly and relatively automatically directed to 1) stimuli with potentially negative implications for the self e.g., [50], 2) stimuli that are emotionally-valenced and thus are associated with greater arousal e.g., [51,52] and 3) stimuli that are novel and distinctive e.g., [53,54]. Therefore, it seems not surprising that the sole presentation of Caucasian facial expressions of emotions elicited stronger amygdala activation in our Asian participants (both females and males) as compared to Caucasian Europeans. Nevertheless, by analyzing the impact of gender of poser we extended current knowledge and future studies might want to further analyze whether this factor also affects studies on gaze perception.

We also observed a significant inverse correlation between duration of stay and amygdala activation during emotion processing in the Asian group that was not mediated by gender of participant. This result further supports our previous finding from an exclusively male sample, reflecting possible adaptation and familiarity effects on the neural level and support the view that the amygdala plays an essential role in the neural network underlying the “alien-effect” and race processing in more general cf. [13,14]. As pointed out by Kitayama and Park [1], experience is powerfully organized by culture and thus we assume that the observed association between duration of stay and amygdala activation reflects neural learning and acclimatization processes that can be measured within the first year and eventually might also improve recognition accuracy. However, no significant correlation emerged between behavioral performance and duration of stay, probably for two reasons: 1) adaptation effects manifest on the neural level before behavioral performance differences can be measured, and 2) the fact that there is no significant association of behavioral performance and duration of stay might be due to the restricted time of residency (max. 1 year) in our inclusion criteria since this was a cross-sectional study. As pointed out by Elfenbein and Ambady [40], a statistically significant improvement in performance was detectable after 2.4 years spent in the foreign culture. Longitudinal studies with several time points would be necessary to clarify after what time adaptation effects can be measured in which neural regions and when neural changes lead to detectable improvements in performance.

### Intracultural gender differences in amygdala activation

Analysis of gender differences in amygdala activation between Asian females and males as well as European females and males revealed no significant gender difference. This finding was corroborated by the result from the whole-brain analysis and is consistent with previous results from others e.g., [26] and recent findings from our own group [31,32] addressing emotion recognition abilities in healthy subjects. Considering Asian gender differences, rarely have studies explicitly addressed this topic in healthy Asian participants and observed no significant difference in amygdala activation [27,28]. Moreover, several studies showed that gender differences in personality, values, and emotions are larger in “Western” cultures. They are seen as a product of self-stereotyping, which occurs when between-gender social comparisons are made which are more likely, and exert a greater impact, in Western nations e.g., [55]. Thus, we believe while gender effects in amygdala activation during emotion processing and more specifically during emotion recognition might occur in clinical samples e.g., [56] in healthy females and males, especially from Asian origin, and using the described task, no such differences will arise cf. [31,32]. Nevertheless, gender differences in amygdala activation during various emotional behavior e.g., humor processing: [57]; empathy: [58,59] have been observed indicating that “Western” females more strongly rely on regions associated with emotion processing, i.e. amygdala, insula, posterior cingulate cortex, while males rather recruit cognition-related areas, i.e. temporo-parietal junction. Here, fMRI studies investigating the interaction of culture and gender on the neural and behavioral correlates are missing but are needed if our understanding is to be extended.

### Emotion recognition performance and the alien-effect

The “alien-effect” was also evident in the emotion recognition performance, as recognition accuracy differed significantly between groups: Asian females and males had greater difficulties with Caucasian expressions of anger and disgust, which was apparent in their behavioral performance during scanning.

Anger is considered to be a very powerful and threatening emotion particularly when depicted by out-group targets cf. [47,60,61] and some studies even suggest that powerful emotions are more intensely experienced and expressed by men, while women rather experience emotions associated with powerlessness, such as fear, sadness and shame e.g., [62]. Despite this gender issue, some cross-cultural studies also indicate that Caucasian expressions of anger are less accurately recognized by Asian participants than by Caucasian subjects, in particular Caucasian Americans e.g., [33,41]. Interestingly, Huang et al. also observed an inter-Asian difference in anger recognition, with Chinese subjects performing significantly better than Japanese subjects when confronted with Japanese and Caucasian expressions of anger. Huang and colleagues thus suggest that 1) Asian raters do not judge negative emotions – irrespective of poser ethnicity – the same way that Caucasians do, and 2) Japanese raters obviously have a stronger tendency not to label negative emotional expressions as negative but tend to rather pick the neutral category. We investigated participants from China (n = 17) and Japan (n = 7) but the small sample size prevented computing of any inter-Asian analysis of performance. Therefore, our results need to be replicated in a larger sample to allow comparison between Asian subjects of different ethnicities. At the moment, our data support the assumption of Huang et al. [33] suggesting that particularly expressions of negative emotions, here anger, might be strongly influenced by so-called nonverbal dialects in expression style. Additionally, behavioral performance and choice of response might also be influenced by cultural stereotypes, in particular by the cultural evaluation of negative emotions.

### Limitations

While this study provides new insight into cultural and gender effects on amygdala activation during emotion recognition, several methodological constraints have to be considered. Since this was a cross-sectional study we cannot make any inferences on the further course of the adaptation process, in particular regarding emotion recognition performance. As pointed out above, we can only speculate that the initial difficulties apparent in the lower recognition accuracy may diminish after a certain time spent in the foreign culture and only longitudinal studies with several time points allow demonstration of neural and behavioral learning processes and the underlying transfer processes.

We were particularly interested in cultural effects on amygdala activation and thus relied on a specifically optimized protocol with restricted brain coverage. However, in light of the significant behavioral differences for anger and previous results on the neural circuits of race processing cf. [14], whole brain coverage might be more favorable to detect differences in higher cortical areas (e.g., insula, anterior cingulate cortex, prefrontal cortex) and enable analysis of effective connectivity of the amygdala within the emotional network. In the future this should be possible using phased-array head coils with improved sensitivity at higher magnetic fields cf. [63].

Moreover, presenting more stimuli per emotion would have allowed emotion specific analysis and thus might have enabled further understanding of recognition difficulties apparent in significantly reduced performance for angry expressions in Asians. Despite the lack of standardized and validated stimuli from Asian subjects in our stimulus material, presentation of these stimuli would have enabled a thorough analysis of in-group and out-group effects. However, we were specifically interested in how faces of the major ethnic group immigrants with a different ethnic background are confronted with are processed on the neural and behavioral level. Cross-cultural studies showing in-group and out-group faces to in-group and out-group raters are scarce, particularly in neuroimaging experiments e.g., [11,15-17,38] and most studies have shown stimuli of different ethnic targets to participants from only one ethnic group or mixed groups, not differentiating between several ethnic groups e.g., [64]. Moreover, Future studies might want to investigate the impact of duration of stay on the neural substrates of the in-group and out-group effects in facial emotion recognition.

More generally, the selection of participants per sé is biased. We refer to Asians but only measured a small group of Chinese and Japanese females and males. Moreover, evidence has accumulated that genetic differences are inextricably intertwined with cultural differences [2,65]. However, in this study we concentrated on the effect of culture on amygdala activity; it was not our aim to highlight the causes of these cultural differences.

## Conclusions

This is the first study to investigate the impact of gender, gender of poser, and ethnic group on emotion recognition and its behavioral and neural correlates in Asian and European females and males. We observed bilateral amygdala activation to emotional expressions in all groups. In the Asian sample, a stronger response of the amygdala was observed bilaterally and paralleled by reduced performance, especially for angry and disgusted faces. ROI analysis revealed no significant gender difference in amygdala activation, but a significant interaction of gender of poser and ethnic group, indicating stronger activation in the Asian sample, particularly for male expressions. Moreover, we observed a significant inverse correlation between duration of stay and amygdala activation indicating that exposure to a foreign ethnic group is a relevant factor for neuroimaging studies addressing emotion processing in female and male immigrants. Particularly in times of globalization and increasing international exchange and interaction, understanding nonverbal communication styles e.g. by accurately recognizing emotional expressions, is critical for successful societal integration and interaction between members of different ethnic groups and this competency underlies adaptation effects. These adaptation effects however might not be limited to emotional competencies, thus the impact of duration of stay might also occur for cognitive functions which needs to be elucidated in studies to come. Notably, in our study, culture and gender of poser significantly modulated emotion recognition accuracy and amygdala activation, indicating that not only between-group differences but also within-group differences, particularly regarding the impact of stimulus material on neural activation, should be considered.

Taken together, this study demonstrates the first attempt to analyze the impact of gender and culture on amygdala activation during emotion recognition. While we observed no gender difference, culture and gender of poser asserted significant effects on the behavioral and neural correlates of this emotional capacity, thereby extending our knowledge on the bases of emotion recognition differences between participants with divergent cultural backgrounds.

## Methods

### Sample

Fourty-eight participants were recruited via advertisements posted at the University of Vienna and the Medical University of Vienna, Austria. The Asian group comprised twelve right-handed healthy females aged 19–32 years (mean age 23.5 years, SD = 3.9) and 12 right-handed males aged 22–35 years (mean age 25.6 years, SD = 3.6). Asian subjects were exchange students from China (n = 17, 8 females) and Japan (n = 7, 4 females). An important inclusion criterion for the Asian subjects was duration of stay: all Asian subjects had been in Europe for less than one year (mean: 5.8 months, SD = 3.4; min: 1 month, max: 12 months) and spoke English fluently. We specifically chose exchange students as this sample is not affected by age effects (being homogeneous and young), has a similar educational background and because these are subjects that visit another country a) for a limited time only, and b) on a voluntary basis.

The Caucasian European group was made up of twelve right-handed healthy females aged 19–29 years (mean age 23.75 years, SD = 2.8) and 12 right-handed males aged 22–34 years (mean age 25.58, SD = 3.3). All subjects were financially reimbursed for their participation and written informed consent was obtained. The study was approved by the local ethics committee and subjects were treated according to the Declaration of Helsinki (1964) regarding treatment of human research participants.

Exclusion of psychiatric disorders (according to DSM-IV) was ascertained by the Structured Clinical Interview (German Version of the SCID, [66]) and the usual exclusion criteria for MRI were applied.

Asians and Europeans were of similar age (F (3,46) = 0.005, p = .942) and had completed comparable number of years of education (F (1,46) = 0.96, p = .444). Alexithymia scores did not differ significantly between the groups (TAS-20, F (1,46) = 0.398, p = .532). In addition, Asian and European subjects did not differ significantly in their estimated nonverbal intelligence (Standard Progressive Matrices [67], F (1,46) = 1.314, p = .258). Moreover, neither a significant gender (all p-values > .185) nor a significant gender-by-ethnic group interaction (all p-values > .334) occurred.

### Functional task

The explicit emotion recognition task used in this study consisted of 30 color photographs of evoked facial expressions portraying the five basic emotions (anger, disgust, fear, happiness and sadness) and an equal number of neutral expressions. All images were taken from a stimulus set which has been standardized and used repeatedly in neuroimaging research (for their development, see [68]). The stimuli were balanced for emotion and gender. Each actor appeared only once and all actors were Caucasians. Since we were interested in minor cultural influences reflected in initial difficulties with regard to emotion recognition in a foreign ethnic group, we did not assess recognition accuracy for in-group faces in the Asian group on the assumption that emotion recognition of in-group facial expressions is a general basic ability which is given in all ethnicities without any significant differences (for review see [40,69]).

Stimulus presentation was randomized with regard to emotion and the order of presentation was kept constant between subjects. Subjects were instructed to choose the correct emotion from two possibilities presented on the left and right of the face by pressing the corresponding button of a response box using the right middle and index finger as quickly as possible. One of the options was correct and the other was selected at random from the other categories (see above). Facial expressions were presented for a maximum of 5 s with a randomized, variable interstimulus interval (ISI) ranging from 12 s to 18 s (during which subjects viewed a scrambled face with a central crosshair). Manual responses triggered immediate progression to the next ISI. Stimuli were projected onto a screen and viewed by the participants via a mirror mounted on the head coil. The presentation of images, recording of responses, and acquisition of scanner triggers (one per TR) was controlled with the Presentation software package (Neurobehavioral Systems, Inc., Albany, CA, USA).

### Behavioral data analysis

Statistical analyses were performed using SPSS 15.0 and unless otherwise specified, the level of significance was p = .05 (two tailed). The behavioral data (i.e., emotion recognition performance and reaction times) acquired during scanning were analyzed with a repeated measures ANOVA, with emotion (anger, disgust, fear, happiness, sadness and neutral) and gender of poser (female vs. male) as within-subject factors and ethnic group (Asian vs. European) and gender (female vs. male) as between-subjects factors.

### FMRI acquisition parameters and data processing

All subjects were examined with a 3 Tesla Medspec whole-body scanner (Bruker Biospin, Ettlingen, Germany) at the MR Centre of Excellence, Medical University of Vienna, Austria. Functional imaging was performed in the axial plane using gradient-recalled echo planar imaging (EPI). Ten oblique axial slices centered on the amygdala were acquired using asymmetric k-space sampling (FOV = 25 × 21cm, matrix size 128 × 91, slice thickness 2 mm, slice gap 0.5 mm, TR = 1000 ms, TE = 31 ms, 570 volumes per run) as has been shown to be sensitive enough to measure reliable amygdala activation [70-73]. Cardiac action and breathing were digitally recorded to allow physiological artifact correction in post-processing which has been shown to increase the sensitivity of fMRI analyses, especially in the amygdala region [74,75].

Functional data were preprocessed using SPM2 (http://www.fil.ion.ucl.ac.uk/spm/spm2.html) to allow direct comparison with the Asian male data [18]. Images were slice timing corrected, realigned to the mean image and normalized into the standardized stereotactic MNI space. Functional data sets were spatially smoothed using an isotropic Gaussian kernel with a full-width-at-half-maximum of 9 mm. For this event-related design, each stimulus was modeled with a separate regressor, based on the individual response period convolved with the canonical hemodynamic response function and its temporal derivative. An additional box-car regressor without hemodynamic delay was used to account for signal changes due to head motion during stimulus presentation. To exclude low-frequency confounds, the data were high-pass filtered using a discrete cosine basis transform set with a cutoff period of 128 s. Regressors of each emotional stimulus were pooled to assess brain responses to emotional expressions, and the same procedure was applied for neutral faces to retrieve brain response to neutral stimuli. Moreover, a contrast including all emotional stimuli minus the neutral expressions was used to separate amygdala activation towards emotional faces vs. neutral expressions. Statistical analysis was performed at the individual and group levels. To detect group differences, contrast images from all subjects were included in a second level random effects analysis. To analyze the impact of gender and culture on whole brain activation, two-sample t-tests were performed comparing Females vs. Males and Asians vs. Europeans. All results are reported at p < .05, FDR corrected.

#### Region of interest (ROI) analysis

Since our main hypothesis focused on the amygdala we performed a ROI analysis with the aim of maximizing the sensitivity to amygdala results. Values for amygdala ROIs were extracted using a template based on the MNI single subject brain [76], as defined in MRIcro (http://www.sph.sc.edu/comd/rorden/template.html). Mean parameter estimates were extracted from SPM results for left and right amygdala ROI in each condition and subject using IDL (Interactive Data Language, RSI Inc., Boulder, CO, USA). An emotion > neutral contrast was calculated, pooling all emotional stimuli minus all neutral stimuli. This subtraction contrast removed the effect of any baseline changes in signal, such as scanner drift. Levene-tests for homogeneity of variances indicated homoscedasticity for the parameter estimates across all subjects (contrast: emotion > neutral for female and male posers separately) of left and right amygdala (left: both p > .340; right: both p > .453). A four-way ANOVA was applied with ethnic group (Asian vs. European) and gender (female vs. male) as between-subjects factor and laterality (left vs. right amygdala) and gender of poser (female vs. male) as within-subject factors. In cases of violation of sphericity assumption Greenhouse-Geisser corrected p-values are presented.

#### Corollary analyses

Correlation analyses were performed between recognition accuracy and right and left amygdala response. To analyze the influence of duration of stay a correlation analysis between months of stay in Austria and behavioral performance as well as amygdala activation was computed for the Asian sample. Moreover, to assess whether gender mediates the correlation between duration of stay and amygdala activation we performed multiple hierarchical linear analyses with amygdala activation as dependent variable and gender and duration of stay as independent variables as suggested by [77].

## Authors’ contributions

BD and UH designed the study, recruited subjects, analyzed behavioral and functional data, and wrote the manuscript. CW and SR were the physicists who conducted the fMRI measurements, helped with functional data analyses and writing of the manuscript. IKE, RCG and EM helped with interpretation of data and final version of manuscript. All authors contributed to and have approved the final manuscript.
